# 2-Deoxyglucose and Beta-Hydroxybutyrate: Metabolic Agents for Seizure Control

**DOI:** 10.3389/fncel.2019.00172

**Published:** 2019-04-30

**Authors:** Jong M. Rho, Li-Rong Shao, Carl E. Stafstrom

**Affiliations:** ^1^Section of Pediatric Neurology, Department of Pediatrics, Alberta Children’s Hospital Research Institute, Hotchkiss Brain Institute, Cumming School of Medicine, University of Calgary, Calgary, AB, Canada; ^2^Department of Clinical Neurosciences, Alberta Children’s Hospital Research Institute, Hotchkiss Brain Institute, Cumming School of Medicine, University of Calgary, Calgary, AB, Canada; ^3^Department of Physiology and Pharmacology, Alberta Children’s Hospital Research Institute, Hotchkiss Brain Institute, Cumming School of Medicine, University of Calgary, Calgary, AB, Canada; ^4^Division of Pediatric Neurology, Department of Neurology, Johns Hopkins University School of Medicine, Baltimore, MD, United States

**Keywords:** epilepsy, 2-deoxyglucose (2DG), glycolysis, ketosis, ketone body, beta-hydroxybutyrate, ketogenic diet, metabolic therapy

## Abstract

Current anti-seizure drugs (ASDs) are believed to reduce neuronal excitability through modulation of ion channels and transporters that regulate excitability at the synaptic level. While most patients with epilepsy respond to ASDs, many remain refractory to medical treatment but respond favorably to a high-fat, low-carbohydrate metabolism-based therapy known as the ketogenic diet (KD). The clinical effectiveness of the KD has increasingly underscored the thesis that metabolic factors also play a crucial role in the dampening neuronal hyperexcitability that is a hallmark feature of epilepsy. This notion is further amplified by the clinical utility of other related metabolism-based diets such as the modified Atkins diet and the low-glycemic index treatment (LGIT). Traditional high-fat diets are characterized by enhanced fatty acid oxidation (which produces ketone bodies such as beta-hydroxybutyrate) and a reduction in glycolytic flux, whereas the LGIT is predicated mainly on the latter observation of reduced blood glucose levels. As dietary implementation is not without challenges regarding clinical administration and patient compliance, there is an inherent desire and need to determine whether specific metabolic substrates and/or enzymes might afford similar clinical benefits, hence validating the concept of a “diet in a pill.” Here, we discuss the evidence for one glycolytic inhibitor, 2-deoxyglucose (2DG) and one metabolic substrate, β-hydroxybutyrate (BHB) exerting direct effects on neuronal excitability, highlight their mechanistic differences, and provide the strengthening scientific rationale for their individual or possibly combined use in the clinical arena of seizure management.

## Key Points

• Cellular metabolism plays a key role in the modulation of neuronal excitability.
• 2-Deoxyglucose (2DG), an inhibitor of glycolysis, abrogates seizure activity and retards epilepsy progression both *in vitro* and *in vivo*.
• Beta-hydroxybutyrate (BHB), a by-product of fatty acid oxidation, exerts both anti-seizure and neuroprotective effects.
• 2DG and BHB offer potential novel avenues for suppressing seizure activity and possibly epileptogenesis.

## Introduction

For decades, the mechanistic focus in the field of epilepsy research has been on ion channels and transporters that regulate neuronal excitability at the synapse. Indeed, virtually all anti-seizure drugs (ASDs) are believed to exert their clinical effects on synaptic targets, whether at the pre- or post-synaptic terminals (Rogawski et al., 2016). However, it has become increasingly clear that metabolic factors can potently affect neuronal excitability and even influence the activity of key cellular membrane-bound ion channels such as inhibitory adenosine and GABA_A_ receptors and ATP-sensitive potassium (K_ATP_) channels (Reid et al., 2014; Bazzigaluppi et al., 2017). The steadily increasing scientific arena of metabolic control of neuronal excitability has been catalyzed by the clinical success and evidence-based validation of the high-fat, low-carbohydrate ketogenic diet (KD) and its variants [i.e., medium-chain triglyceride (MCT), modified Atkins diet (MAD), and low-glycemic index treatment (LGIT)] (Neal et al., 2008; Rho and Stafstrom, 2012; Lutas and Yellen, 2013; Gano et al., 2014). While the fundamental mechanisms underlying the efficacy of these metabolic treatments across numerous epileptic conditions remain unclear, there is growing evidence that specific metabolic substrates and enzymes may act directly or indirectly to induce anti-seizure (and even neuroprotective) effects. Moreover, substrate or pharmacological modulation of key biochemical pathways has shown similar benefits (Rogawski et al., 2016).

## Inhibition of Glycolysis

The KD originated from the centuries-old observation that fasting led to seizure control and was thus formulated to mimic the physiological effects of fasting (Gano et al., 2014). Clinicians also noted that seizure control while on the KD could be abruptly lost when carbohydrates, which would break the ketosis, were ingested (Huttenlocher, 1976). This observation led to the general hypothesis that carbohydrate restriction alone might protect against seizure activity (Greene et al., 2003). Further amplifying this idea was the growing notion that reducing total calorie intake *per se* could also suppress seizures and provide neuroprotection (Greene et al., 2003; Ingram and Roth, 2011; Yuen and Sander, 2014; Pani, 2015).

Glucose is an obligate energy source for the brain, which is a highly energy-dependent organ, consuming approximately 20% of the body’s total caloric requirements at rest (Magistretti and Allaman, 2015). Seizure activity places further demands on the overall brain metabolic milieu due to excessive neuronal activity – reflected by the aberrant high-voltage activity seen from single neurons to brain networks using microelectrodes and extracellular field and surface scalp electrodes. Neurometabolic coupling during seizure activity not only depends on energy metabolism of neurons, but may also involve astrocytes as they may provide neurons with fuel (i.e., lactate) through the lactate shuttle (Cloix and Hévor, 2009; Magistretti and Allaman, 2015; Boison and Steinhäuser, 2018, but see Dienel, 2017). In addition, brain microvasculature integrity is of paramount importance in supporting the neurometabolic fluctuations required to enable neuronal excitability (Librizzi et al., 2018). Not surprisingly then, deficits in glucose availability and usage have been linked to several neurological disorders (Mergenthaler et al., 2013). By contrast, enhanced neuronal activity, such as during epileptic seizures, significantly increases regional blood glucose utilization, as shown by human positron emission tomography (PET) studies (Cendes et al., 2016), thus suggesting a rationale for potential seizure control through metabolic interventions.

## 2-Deoxyglucose, A Glycolysis Inhibitor

As mentioned above, the KD mimics fasting in restricting the intake of the main source of brain energy (i.e., carbohydrates) while supplying fat and protein to generate ketone bodies as an alternative energy source. While the mechanisms of seizure control by the KD are likely to be multi-faceted (Kawamura et al., 2016), it is important to note that the KD bypasses glycolysis, and an intake of even a small amount of sugar quickly reverses its otherwise seizure-stabilizing effects (Huttenlocher, 1976). This suggests that energy production by glycolysis may be important for seizure activity and bypassing or suppressing glycolysis may represent a key mechanism involved in KD treatment. Collectively, these observations provide the rationale for the notion that inhibitors of glycolysis may mimic in part the therapeutic effects of the KD. It is also well known that ketolysis itself decreases glycolytic flux, and it has been proposed that ketone bodies attenuate neuronal cellular excitability through this mechanism (Lutas and Yellen, 2013). As there are known agents that restrict glycolytic flux, this overarching hypothesis is eminently testable.

**Figure 1:**
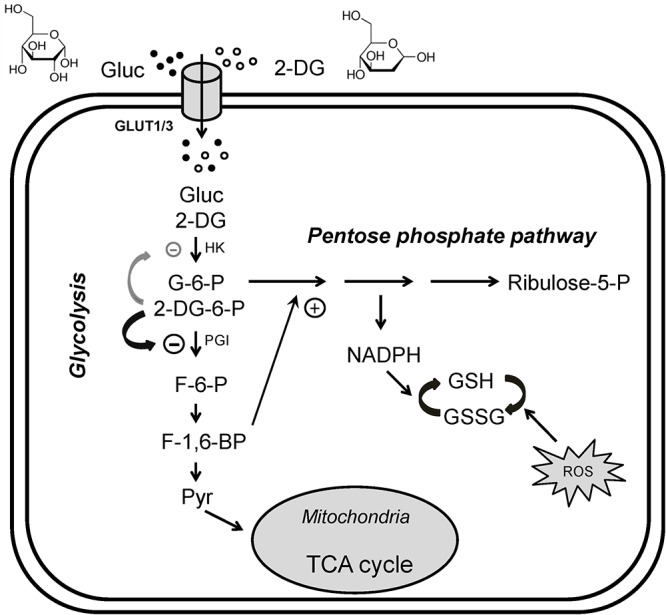
Glycolytic inhibition via 2-deoxyglucose (2DG). 2DG decreases glycolytic flux, reduces cell energy production and produces anti-seizure effects. Glucose (Gluc) and 2DG enter the cytoplasm via glucose transporters (GLUT3 in neurons or GLUT1 in glia). During glycolysis, glucose is first phosphorylated to glucose-6-phosphate (G-6-P), which is then converted into fructose-6-phosphate (F-6-P). F-6-P is converted into fructose-1,6-bisphosphate (FBP) and ultimately to pyruvate via multiple steps. Pyruvate is the final product of glycolysis; it enters mitochondria to participate in the tricarboxylic acid (TCA) cycle for oxidative ATP production. G-6-P also enters the pentose phosphate pathway (PPP), generating reduced nicotinamide adenine dinucleotide phosphate (NADPH) and glutathione (GSH), which attenuates cell damage caused by reactive oxygen species (ROS). In addition, GSH may have antiseizure properties. Once inside the cell, 2DG is phosphorylated to 2DG-6-P, which cannot be further metabolized. 2DG-6-P is trapped and accumulates in the cell, competitively inhibiting phosphoglucose isomerase (PGI, primary effect) and hexokinase (HK), limiting the conversion of G-6-P to F-6-P and glucose to G-6-P, two of the rate-limiting steps in glycolysis. Increased FBP (e.g., by exogenous administration) may also enhance PPP activity and produce an antiseizure effect (plus sign). The chemical structures of glucose and 2DG are indicated at the top of the figure. Modified from Shao et al. (2018), with permission from Epilepsia Open.

One promising glycolysis inhibitor for seizure protection is the glucose analog 2-deoxyglucose (2DG) which differs from glucose by the substitution of oxygen from the 2′ position (Figure 1). Similar to glucose, 2DG is transported into cells and is phosphorylated to 2DG-6-phosphate at the 6′ position by hexokinase (HK), but this phosphorylated substrate cannot be converted to fructose-6-phosphate by phosphoglucose isomerase (PGI), and is thus trapped in the cell. The accumulation of 2DG-6-phosphate competitively inhibits the rate-limiting enzymes, primarily PGI (Wick et al., 1957) but also HK (Pelicano et al., 2006), hence partially blocking glycolysis. In addition, inhibition of PGI would divert glycolysis to the pentose phosphate pathway (PPP), producing ribulose and glutathione. It should be kept in mind that 2DG, like glucose, is not only taken up by neurons (via glucose transporter 3) but is also taken up by glial cells (via glucose transporter 1), inhibiting astrocytic glycolysis. Recent studies hypothesize that astrocytes may transport their glycolytic end-product, lactate, as an alternative fuel source to neurons through the “astrocyte-neuron lactate shuttle” (ANLS) (Pellerin and Magistretti, 1994, but see Dienel, 2017). Therefore, 2DG may potentially affect neuronal activity indirectly by suppressing astrocytic glycolysis. This biochemical feature has been successfully exploited to identify energetically active cells, notably hyperexcitable brain cells or rapidly dividing cancer cells (Pelicano et al., 2006; Cheong et al., 2011). Cancer cells, even in aerobic conditions, tend to use glycolysis for energy production over oxidative phosphorylation (Warburg effect); 2DG enhances oxidative phosphorylation, countering the Warburg effect (de Padua et al., 2017). For decades, fluorinated 2DG (^18^F-2DG) has been safely used as a tracer to reveal regional glucose utilization by PET (Wree, 1990; Cendes et al., 2016).

With respect to epilepsy, 2DG has been shown to exert broad activity in several *in vitro* and *in vivo* models of acute seizures (Stafstrom et al., 2009). *In vitro*, 2DG attenuates high-frequency epileptiform bursting in the high extracellular potassium model, and in response to two chemoconvulsants – 4-aminopyridine (4-AP, a non-selective potassium channel blocker) and bicuculline [a γ-aminobutyric acid (GABA_A_) receptor antagonist] (Stafstrom et al., 2009). 2DG was also effective in hippocampal brain slices whether synaptic activity in CA3 pyramidal neurons was left intact or blocked by AMPA, NMDA, and GABA_A_ receptor antagonists (DNQX, APV, and bicuculline, respectively) (Shao and Stafstrom, 2017). Interestingly, CA3 neuronal firing persisted even after intracellular administration of 2DG, suggesting that glycolytic inhibition of individual neurons is not sufficient to stop neuronal firing. Indeed, bath application of 2DG in hippocampal slices abolished epileptiform bursts induced by a Mg^2+^-free medium containing 4-AP in a dose-dependent manner (Shao and Stafstrom, 2017). Collectively, these results indicate that 2DG can markedly block epileptiform activity in both interictal and ictal states. Recently, however, a study reported that 2DG reduced Cs^+^- and bicuculline-induced interictal-like activity while increasing ictal-like activity in rat hippocampal slices (Nedergaard and Andreasen, 2018). The precise reasons for this discrepancy are unclear. Possibly, in most of the aforementioned *in vitro* seizure models, either K^+^ channel or GABAergic inhibition is unaffected, whereas in the model used by Nedergaard and Andreasen (2018), both inhibitory mechanisms are blocked, which may hamper seizure termination.

The anti-seizure effects of 2DG were also demonstrated in different types of animal models of seizures. 2DG increased the mean after-discharge threshold in the rat perforant path kindling model (Garriga-Canut et al., 2006; Stafstrom et al., 2009). Further, 2DG blocked seizure activity in the 6-Hz corneal stimulation model (Barton et al., 2001; Stafstrom et al., 2009; Gasior et al., 2010) and in Frings audiogenic seizure-susceptible mice (Stafstrom et al., 2009). In addition, 2DG significantly delayed seizure onset and diminished seizure severity and duration in the pilocarpine model, and delayed seizure onset in the kainate (KA) model (Lian et al., 2007). In contrast, 2DG was unable to protect against seizures induced by pentylenetetrazol (PTZ) or maximal electroshock (MES) (Gasior et al., 2010). While 2DG’s spectrum of activity was not universal in some standard experimental models, its profile has certainly been shown to be unique compared to existing anti-seizure medications. Another important aspect of 2DG function deserves mention – 2DG is mostly taken up by cells that are metabolically active. This use-dependence affords a distinct advantage for any therapeutic strategy aimed at targets in brain areas exhibiting hyperexcitable and hypersynchronous discharge, as seen with epileptic seizures.

The acute and chronic effects of 2DG in different model systems are likely due to distinct cellular and molecular mechanisms. With respect to anti-epileptogenesis as reflected in the rat kindling model, the most intriguing mechanism reported is 2DG’s actions in decreasing brain-derived neurotrophic factor (BDNF) and its receptor trkB (Garriga-Canut et al., 2006), both of which are necessary for kindling to occur (He et al., 2004). Specifically, these changes are mediated by the transcriptional repressor Neuron Restrictive Silencing Factor (NRSF) and its co-repressor, the nicotinamide adenine dinucleotide hydride (NADH)-sensitive carboxy-terminal binding protein (CtBP), which together target the promoter regions of both *Bdnf* and *TrkB* genes (Garriga-Canut et al., 2006). There is growing evidence that repression of *BDNF* and *trkB* retards kindling progression, and this may be the key mechanism underlying the anti-epileptogenic effects of 2DG (Garriga-Canut et al., 2006; Huang and McNamara, 2006; Hu et al., 2011). An additional mechanism implicated in 2DG action is upregulation of mRNAs of the ATP-sensitive potassium (K_ATP_) channel subunits Kir6.1 and Kir6.2 (Yang et al., 2013). Plasmalemmal K_ATP_ channels, when open and thus allowing potassium efflux, hyperpolarize the membrane. 2DG may also modulate GABAergic signaling, specifically by potentiating extra-synaptic tonic GABAergic currents through activation of neurosteroidogenesis (Forte et al., 2016).

Other than its anti-seizure activity, 2DG has also been shown to be neuroprotective. Specifically, 2DG protected against the deleterious oxidative and metabolic effects of kainic acid in rat hippocampal cell cultures by enhancing stress responses (Lee et al., 1999) and reduced seizure occurrence in the mouse bilateral carotid artery occlusion model (Redjak et al., 2001). These neuroprotective actions may result from possible enhancement of PPP flux which provides endogenous antioxidant glutathione, but also appear to involve mechanisms distinct from direct glycolytic restriction, including reduced oxidative stress through ketogenesis and sustaining mitochondrial function (Yao et al., 2011) and activation of adenosine monophosphate (AMP)-activated protein kinase which confer resistance to oxidative stress (Park et al., 2009).

Notably, other researchers reported that 2DG might exert pro-seizure/pro-epileptogenic effects under certain conditions, which adds to the complexity of 2DG action. One study observed that the latency to seizure onset was decreased after 2DG administration in the PTZ and kainate models (but prolonged in the 6 Hz model) (Gasior et al., 2010). While the mechanism(s) underlying these findings remain(s) unclear, 2DG is known to increase cerebral blood flow (Elman et al., 1999), so it is possible that seizure activity might be promoted acutely as initial neuronal and network excitability is fueled, which may confound the interpretation of these findings. More recently, it was reported that daily intracerebroventricular administration of 2DG for 4 weeks resulted in spontaneous seizures in two of ten rats and a depolarized GABA equilibrium potential (E_GABA_) suggesting a pro-epileptogenic effect, although the acute effects of 2DG were more complex (i.e., decreased synaptic excitability but depolarized membrane potential and E_GABA_) (Samokhina et al., 2017). It is not known what underlies the discrepancies seen with 2DG in different seizure models, and between acute and chronic treatment protocols with 2DG. Clearly, more studies are needed to resolve these differences and to further understand the effects of 2DG, and more broadly metabolic interventions for seizures/epilepsy.

## Ketone Bodies as Anti-Seizure Agents

While the efficacy of the KD in patients with medically intractable epilepsy is no longer debatable (Freeman et al., 1998; Neal et al., 2008, 2009; Lambrechts et al., 2017), the mechanisms underlying its clinical effects remain unclear. Many hypotheses have been advanced, including but not limited to favorable changes in neurotransmitter systems (e.g., GABA, glutamate, and adenosine), glycolytic restriction/diversion, enhancement of tricarboxylic acid (TCA) cycle function, improved cellular bioenergetics and mitochondrial function (with decreased oxidative stress), and direct inhibitory effects of fatty acids (Rogawski et al., 2016).

The hallmark feature of the KD is the production of ketone bodies (KB) [i.e., β-hydroxybutyrate (BHB), acetoactate (ACA) and acetone] through fatty acid oxidation primarily in the liver, and possibly the brain (Aalling et al., 2018). Ordinarily, fatty acids are converted to acetyl-CoA which then enters the TCA cycle. However, when fatty acid levels rise and exceed maximal TCA cycle capacity – as in the case with KD treatment – acetyl-CoA is shunted to ketogenesis. Acetoacetyl-CoA is produced from two acetyl-CoA molecules through a thiolase enzyme and is then is used for the synthesis of ACA which is interconverted with BHB through a dehydrogenase enzyme. Acetone is a spontaneous decarboxylation product of ACA and is eliminated primarily through the lungs and kidneys. BHB and ACA are transported to the brain interstitial space via monocarboxylic acid transporters (MCTs) and enter mitochondria which convert these ketone bodies to acetyl-CoA through several enzymatic steps. The reconstituted acetyl-CoA species are then utilized by the TCA cycle for ATP production. Increased ATP production can then lead to further biochemical and physiological effects that can contribute to seizure control and neuroprotection (see below).

In this regard, a persistent question has been whether ketone bodies are direct mediators; that is, do ketone bodies exert biological effects that attenuate neuronal hyperexcitability and/or synchrony, beyond their classic role as fuel for ATP production? The common belief is that they do not, based on clinical studies demonstrating that blood levels of ketone bodies do not correlate well with seizure control (Gilbert et al., 2000; van Delft et al., 2010). Additionally, the mechanistic role for KB is most seriously challenged when considering the low-glycemic index therapy (LGIT) which is an effective treatment for intractable forms of epilepsy but is not associated with significant elevations in blood ketone levels (Pfeifer and Thiele, 2005). Thus, the LGIT must work through distinct mechanisms that have yet to be fully elucidated but may involve K_ATP_ channels and adenosine receptors (Ma et al., 2007; Kawamura et al., 2010).

At least in experimental models, ketosis does not appear to be necessary for seizure control (Thavendiranathan et al., 2000; Dallérac et al., 2017). For example, a MCT KD was unable to block seizures induced by MES, threshold electroconvulsive shock, threshold PTZ and maximal PTZ, despite significant elevations in blood ketone levels (Thavendiranathan et al., 2000). Moreover, a nutritional strategy targeting specific amino acids, fatty acids and carbohydrates constituting a low ketogenic ratio (i.e., the ratio of fat to carbohydrate plus protein by weight) without significant ketosis still resulted in seizure protection in the kainic acid mouse model of epilepsy (Dallérac et al., 2017). It should be noted that the Dallérac et al. (2017) study utilized MCTs (i.e., decanoate) that are known to directly block α-amino-3-hydroxy-5-methyl-4-isoxazolepropionic acid (AMPA) receptors (Chang et al., 2016). Thus, while various dietary approaches may all work to control epileptic seizures, the underlying mechanisms are likely distinct, making it somewhat difficult to compare these with the classic or even modified KD.

Notwithstanding these clinical and laboratory observations, more recent clinical data suggest otherwise (Kim et al., 2015; Buchhalter et al., 2017) and the growing mechanistic properties of ketone bodies strengthen the biological plausibility that these substrates afford both structural and functional neuroprotection in the epileptic brain. In a prospective pilot clinical study of children with medically intractable epilepsy who were treated with the KD, blood BHB levels determined by tandem mass spectrometry were inversely related to seizure frequency (Buchhalter et al., 2017). This is consistent with an earlier report that blood ketone levels (i.e., BHB) correlated better with seizure control than urine ketone levels (i.e., ACA) (Gilbert et al., 2000).

The earliest laboratory reports of ketone bodies exerting anti-seizure effects came in the early 1930s, shortly after the introduction of the KD in clinical practice (Keith, 1931, 1932, 1933, 1935). Specifically, ACA was shown to protect against seizures induced by thujone, a constituent of wormwood oil later shown to be an antagonist of GABA_A_ receptors (Höld et al., 2000). Decades later, acetone was shown to provide dose-dependent beneficial effects in the MES, subcutaneous PTZ, amygdala kindling, and the AY-9944 (an inhibitor of cholesterol biosynthesis, which evokes atypical absence seizures) models of induced seizures or epileptogenesis (Likhodii et al., 2003). Further, in support of Keith’s original studies, ACA was effective in blocking seizures in a model of sensory-evoked reflex seizures, the Frings audiogenic seizure-susceptible mice (Rho et al., 2002). More recently, several research teams demonstrated that BHB, when administered acutely or chronically, conferred seizure protection in immature rat pups against flurothyl-induced (Minlebaev and Khazipov, 2011) and in the rat betamethasone-NMDA model of infantile spasms (Yum et al., 2015). In another study, using the clinically relevant *Kcna1*-null mouse model of developmental limbic epilepsy (that lacks the delayed rectifier voltage-gated potassium channel α subunit), Kim et al. (2015) found that BHB afforded the same degree of seizure control as the full KD. Taken together, these and other studies support the hypothesis that KB can themselves induce anti-seizure effects separate from their role in ATP production. Hence, the possibility that these compounds contribute directly to the clinical effects of the KD cannot be readily dismissed.

So, what are the key molecular targets of ketone body action? As KB were historically viewed as metabolic substrates and not direct modulators of synaptic function, it was not surprising that low millimolar concentrations of BHB and ACA did not influence the activity of GABA_A_ receptors, ionotropic glutamate receptors, or voltage-gated sodium channels in normal rodent hippocampus (Thio et al., 2000; Yang et al., 2007), in contrast to our current understanding of how most of clinically approved ASDs work (Rogawski et al., 2016). However, in a more elegant study, it was shown that neuronal excitability could be suppressed by ketone bodies through a pre-synaptic mechanism (Juge et al., 2010). Specifically, BHB and ACA inhibited the presynaptic release of glutamate by directly competing with Cl^-^ at the allosteric modulation site on vesicular glutamate transporters (VGLUTs). These investigators also demonstrated that the proconvulsant effects of 4-AP, accompanied by presynaptic release of glutamate, could be attenuated by ACA (Juge et al., 2010).

The first mechanistic insights into KB action, however, came from neurochemical experiments in the 1990s. Erecinska et al. (1996) and Daikhin and Yudkoff (1998) demonstrated that ketone bodies (i.e., BHB) altered glutamate metabolism within the tripartite neurovascular unit, comprising neurons, astrocytes and the capillary microvasculature. In the ketotic state, the aspartate-to-glutamate ratio is reduced based on a change in the equilibrium of the aspartate aminotransferase reaction. As the transamination of glutamate to aspartate is decreased, glutamate decarboxylation to GABA is increased (Erecinska et al., 1996; Daikhin and Yudkoff, 1998). Thus, increased synaptic levels of GABA would enable increased inhibitory neurotransmission and dampen seizure activity. While this has long stood as a rational and intuitive mechanistic argument, supported by predicted neurochemical changes in certain brain regions but not the key seizure-prone areas such as the neocortex or hippocampus, more direct evidence has not been forthcoming (Yudkoff et al., 2001; Lund et al., 2009, 2011; Valente-Silva et al., 2015; Zhang et al., 2015). Further, the GABAergic hypothesis of ketone body action doesn’t explain why the KD would be effective in blocking seizure activity in patients with epilepsy who were refractory to GABAergic medications (Freeman et al., 2006).

A generation ago, it was speculated that K_ATP_ channels might be an important link between cellular energy metabolism and plasmalemmal membrane excitability (Schwartzkroin, 1999). Indeed, it has long been known that K_ATP_ channels are activated when the intracellular ATP-to-ADP ratio falls, resulting in efflux of potassium and hence causing membrane hyperpolarization. However, it was not until much later that Ma et al. (2007) demonstrated that ketone bodies influence the activity of K_ATP_ channels. Using brain slices from normal and genetically engineered mice that lack K_ATP_ channels, they showed that BHB and ACA decreased the spontaneous firing of GABAergic interneurons in the substantia nigra pars reticulata (a subcortical modulator of seizure propagation), and that this effect was dependent on K_ATP_ channels and GABA_B_ receptors (Ma et al., 2007). The central thesis has been that ATP levels subjacent to cell membrane-bound K_ATP_ channels are depleted due to heightened neuronal excitability during seizure activity which requires energy, and this would lead to activation of inhibitory K_ATP_ channels.

Despite its intrinsic appeal, there is yet no direct confirmation of this mechanistic hypothesis. Moreover, the KD and ketone bodies have been shown to increase cellular levels of ATP via enhanced mitochondrial biogenesis and respiration (DeVivo et al., 1978; Bough et al., 2006; Kim et al., 2010). Another explanation for ketone body action on K_ATP_ channels was provided by Kawamura et al. (2010) who showed that low-glucose conditions lead to opening of pannexin channels and efflux of ATP through these channels on CA3 pyramidal neurons, subsequent conversion of ATP to adenosine via extracellular ectonucleotidases, and activation of adenosine A1 inhibitory receptors, which are coupled to K_ATP_ channels (Kawamura et al., 2010). Thus, BHB might induce a form of autocrine regulation by enhancing ATP synthesis, with one downstream consequence being cellular membrane inhibition.

Other novel targets for ketone body action have been described in recent years. In the study by Kim et al. (2015) mentioned above, BHB-induced block of spontaneous recurrent seizures in *Kcna1*-null mice was shown to be mediated by an interaction with the mitochondrial permeability transition (mPT) pore, which when activated triggers the release of calcium and pro-apoptotic factors and eventual cell death (Izzo et al., 2016). Previous reports indicated that BHB exerts multiple effects on mitochondria, including increased mitochondrial respiration, enhanced NADH oxidation and ATP production, and reduced reactive oxygen species (ROS) production, all of which either singly or in combination would raise the threshold for mPT (Maalouf et al., 2007; Maalouf and Rho, 2008; Kim et al., 2010). Intriguingly, this was the first demonstration that inhibition of mPT could result in anti-seizure effects.

**Figure 2:**
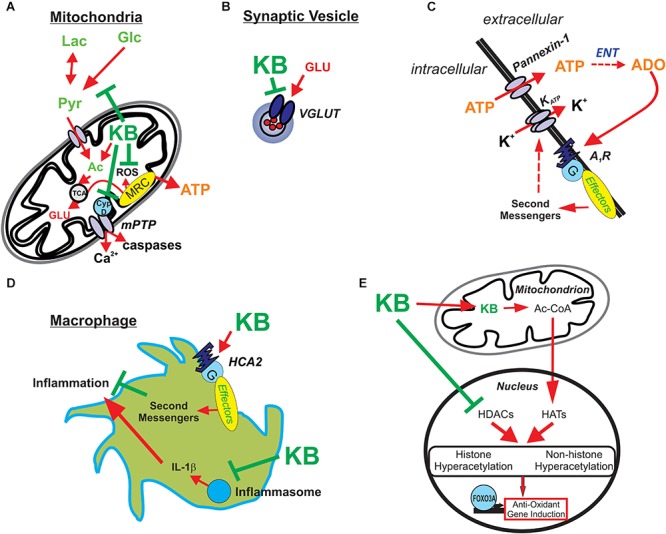
Potential mechanisms underlying ketone body-mediated attenuation of CNS hyperexcitability and neuroprotection. **(A)** Ketone bodies (KB) may enhance ATP production by providing acetyl-CoA (Ac) and inhibiting production of reactive oxygen species (ROS) and the mitochondrial permeability transition (mPT) pore, thereby protecting the cell against oxidative injury and preventing excessive release of calcium. **(B)** KB may inhibit vesicular glutamate transporters (VGLUT), decreasing the amount of glutamate loaded in vesicles and reducing the size of glutamate quanta released during synaptic transmission. **(C)** KB-mediated increases in intracellular ATP and subsequent release through pannexin channels lead to adenosine (ADO) synthesis via ectonucleotidases (ENT) in the extracellular space. ADO in turn binds to inhibitory adenosine type 1 receptors (A1Rs) which are coupled to the indirect opening of K_ATP_ channels. **(D)** KB activates HCA2 receptors and inhibit the assembly of the NRLP3 inflammasome; thus, KB attenuate inflammatory mediators produced by infiltrating macrophages. **(E)** KB also promote histone and non-histone hyperacetylation by increasing acetyl-CoA, a substrate for histone acetyltransferases (HATs), and directly inhibiting histone deacetylases (HDACs) – with the net result of increasing endogenous anti-oxidants (among other actions). Reprinted with permission from Simeone et al. (2018).

There have been a few additional seminal studies showing the pleiotropic activity of ketone bodies, and while these novel targets were revealed in experimental systems outside the context of epileptic tissues, they nevertheless highlight important molecular actions that have become increasingly relevant in epilepsy research. First, BHB was shown to inhibit histone deacetylases (HDACs) in renal tissue, which resulted in increased resistance to oxidative stress via transcription of antioxidant genes regulated by FOXO3A (Shimazu et al., 2013). The relevance of HDACs in epilepsy is underscored by the fact that the broad-spectrum ASD valproic acid is in part an HDAC inhibitor (Monti et al., 2009). Further, BHB’s neuroprotective properties stem from (among other actions) an interaction with the hydroxycarboxylic acid 2 (HCA2) receptor (or GPR109A, also known as NIACR1 or the niacin receptor 1, and encoded by the *HCAR2* gene), a G protein-coupled receptor found on adipocytes, neutrophils, tissue macrophages, and in the anterior cingulate cortex (Rahman et al., 2014). The net effect of BHB binding to HCA2 is a profound anti-inflammatory effect. BHB also targets other key components of the innate immune system. Specifically, it has been shown that BHB prevents the assembly of the innate immune sensor NOD-like receptor protein 3 (NLRP3) inflammasome, a multiprotein complex that induces caspase-1 activation and release of the pro-inflammatory cytokines IL-1β and IL-18 in macrophages (Youm et al., 2015). As neuroinflammation is now unquestionably linked to seizure genesis and epileptogenesis (Vezzani et al., 2015), strategies to mitigate aberrant brain inflammation would be expected to be both anticonvulsant and neuroprotective. Indeed, BHB possesses anti-inflammatory properties, consistent with the fact the KD is highly effective against neuroinflammation-induced epilepsy such as FIRES (febrile infection-related epilepsy syndrome) (Nabbout et al., 2010). A mechanistic summary of ketone body action illustrated in Figure 2.

## 2-Deoxyglucose Versus/Plus Ketone Bodies

From the foregoing discussion, it is important to note both 2DG and the KD can suppress seizure activity but likely exert their effects through distinct yet overlapping mechanisms (Table 1). Ketone bodies have also been shown to possess anti-seizure properties, albeit in preclinical models (Simeone et al., 2018). One major distinction between 2DG and the KD is the fact that 2DG does not induce ketosis. And while 2DG and the KD both block seizures in the 6-Hz corneal stimulation and Frings audiogenic seizure susceptible models, 2DG is ineffective in the MES test which models focal-onset seizures with secondary generalization (Hartman et al., 2008). Further, despite different induction protocols, the progression of kindling is delayed by both the KD and 2DG (Hori et al., 1997; Garriga-Canut et al., 2006; Stafstrom et al., 2009). Thus, while seizure susceptibility is modified by both 2DG and the KD (also ketone bodies) through modulation of biochemical pathways involved in energy metabolism, these interventions do not possess the same anti-seizure profile and cannot be directly compared. That said, the fact that the mechanistic profiles of 2DG and ketone bodies suggest that a combined approach may yield either additive or synergistic effects, especially since a broader spectrum of mechanistic targets and pathways would be influenced. Also, it will be interesting to assess the combined efficacy of 2DG and BHB on seizure control in other chronic epilepsy models with spontaneous seizures such as the pilocarpine and kainate models.

**Table 1:** Mechanistic comparison between 2-deoxyglucose (2DG) and β-hydroxybutyrate.

	Action and target	References
**2-Deoxyglucose**
	Inhibition of phosphoglucose isomerase	Wick et al., 1957
	Inhibition of hexokinase	Pelicano et al., 2006
	Decreased expression of BDNF and trkB	Garriga-Canut et al., 2006
	Upregulation of K_ATP_ channels	Yang et al., 2013
	Increased tonic GABA inhibition via increased neurosteroidogenesis	Forte et al., 2016
	Reduced oxidative stress	Lee et al., 1999; Yao et al., 2011
	Activation of AMP-kinase	Park et al., 2009
	Disruption of glycosylation	Zhang et al., 2014
**β-Hydroxybutyrate**		
	Increased mitochondrial biogenesis and ATP production	DeVivo et al., 1978; Bough et al., 2006; Kim et al., 2010
	Augmented presynaptic synthesis of GABA	Erecinska et al., 1996
	Inhibition of VGLUT release	Juge et al., 2010
	Indirect activation of K_ATP_ channels	Ma et al., 2007
	Reduced oxidative stress	Maalouf et al., 2007; Shimazu et al., 2013
	Raising the threshold for mPT activation	Kim et al., 2015
	HDAC inhibition	Shimazu et al., 2013
	Activation of HCA2 receptors	Rahman et al., 2014
	Prevention of NLRP3 inflammasome assembly	Youm et al., 2015

*Abbreviations: BDNF, brain-derived neurotrophic factor; trkB, Ttropomyosin receptor kinase B; K_*ATP*_ channel, ATP-sensitive potassium channel; GABA, γ-aminobutyric acid; AMP-Kkinase, adenosine monophosphate kinase; VGLUT, vesicular glutamate transporter; mPT, mitochondrial permeability transition; HDAC, histone deacetylase; HCA2, hydroxycarboxylic acid receptor 2; NLRP3, NOD-like receptor protein 3*.

Before formulations of 2DG or BHB are approved for clinical use in patients with epilepsy, efficacy and safety need to be clearly demonstrated. In this regard, the relative safety profile of 2DG has already been established in cancer trials (Pelicano et al., 2006), and epilepsy clinical trials are in progress (Bialer et al., 2017). The half-life of 2DG in humans is estimated to be 5–7 h, so this therapy would be potentially useful with twice-daily dosing (Gounder et al., 2012). With respect to BHB, ketone esters have been investigated preclinically as potential therapeutic agents, primarily due to their ability to produce prolonged elevations in blood ketone bodies (D’Agostino et al., 2013). However, the pharmacokinetics of BHB have not been carefully studied in humans. While ketone esters have yet to be studied in humans with epilepsy, there is already a medical formulation of MCTs that induces significant ketosis in humans (Henderson et al., 2009), and various ketone salts, esters and oils are already widely available through commercial sources and through the Internet. In addition, several metabolites or metabolic strategies have been reported to exhibit potent anticonvulsant actions via alternative mechanisms, including fructose-1,6-bisphosphate (Lian et al., 2007; Shao et al., 2018), decanoic acid (Chang et al., 2016), inhibition of lactate dehydrogenase (Sada et al., 2015), D-leucine (Hartman et al., 2015), and polyunsaturated fatty acids (Dustin and Stafstrom, 2016), all of which are worthy of further exploration for epilepsy therapeutics.

## Conclusion

Metabolic control of neuronal excitability has increasingly become a worthy scientific paradigm for therapeutic development in the field of epilepsy, impacting key mechanisms in seizure genesis and possibly epileptogenesis. The origin and evolution of this approach have been inspired by the clinical success of the KD and its variants. Within this framework, 2DG and BHB represent two promising approaches for seizure control, the former primarily through partial inhibition of glycolysis and downstream transcriptional regulation, and the latter via pleiotropic actions at mitochondrial, epigenetic and cellular levels. While 2DG and BHB each possesses unique preclinical profiles, both have been shown to reduce seizure susceptibility in several animal models and in multiple *in vitro* cellular systems.

However, clinical implementation of metabolism-based approaches such as the KD is not without pragmatic challenges regarding administration and patient compliance. Thus, there is a strong need to identify specific metabolic substrates and/or enzymes that might induce clinical benefits comparable to the KD as a necessary condition for rational experimental therapeutics and later clinical validation. 2DG and BHB may only represent component pieces of diet-based treatments for epilepsy, but together, since they recapitulate two core features of the KD (i.e., glycolytic restriction and products of increased fatty acid oxidation), they could in time help validate the concept of a “diet in a pill” (Rho and Sankar, 2008), or polypharmacy in a pill. Of course, the KD induces far broader mechanistic effects than either 2DG or BHB alone (Rogawski et al., 2016), but the current major advantage of these substrates is that they can already be delivered in palatable forms to patients with epilepsy.

## Author Contributions

JR, CS, and L-RS wrote the manuscript.

## Conflict of Interest Statement

We confirm that we have read the journal’s position on issues involved in ethical publication and affirm that this report is consistent with those guidelines. JR has served as a paid consultant for Accera Pharma, Xenon Pharmaceuticals, Danone Nutricia, and Ajinomoto USA. CS has served as a paid consultant for Mallinckrodt and has received speaker fees from Nutricia. JR and CS have received royalties from UpToDate for a chapter about the mechanisms of seizures and epilepsy. CS is an inventor on a patent application for the use of 2DG as a clinical antiseizure medication through theWisconsin Alumni Research Foundation. The remaining author declares that the research was conducted in the absence of any commercial or financial relationships that could be construed as a potential conflict of interest.
